# Blockade of persistent colored isomer formation in photochromic 3*H*-naphthopyrans by excited-state intramolecular proton transfer

**DOI:** 10.1038/s41598-022-23759-9

**Published:** 2022-11-10

**Authors:** Błażej Gierczyk, S. Shaun Murphree, Michał F. Rode, Gotard Burdzinski

**Affiliations:** 1 Faculty of Chemistry, Adam Mickiewicz University in Poznań, Uniwersytetu Poznańskiego 8, 61-614 Poznań, Poland; 2 Department of Chemistry, Allegheny College, 520 North Main Street, Meadville, PA USA; 3 Institute of Physics, Polish Academy of Sciences, Aleja Lotników 32/46, 02-668 Warsaw, Poland; 4 Faculty of Physics, Adam Mickiewicz University in Poznań, Uniwersytetu Poznańskiego 2, 61-614 Poznań, Poland

**Keywords:** Photochemistry, Physical chemistry, Chemical physics, Excited states, Reaction kinetics and dynamics, Theoretical chemistry, Reaction mechanisms

## Abstract

In photochemistry the excited-state intramolecular proton transfer process (ESIPT) is often observed as a highly efficient singlet excited state depletion pathway, which in the presence of a strong intramolecular hydrogen bond may proceed on a subpicosecond time scale. The present work describes the suppression of unwanted *transoid-trans* isomer formation in photochromic 3*H*-naphthopyran derivatives by the introduction of a 5-hydroxy substituent. According to time-resolved spectroscopy experiments and excited-state ab initio calculations, *transoid-cis* → *transoid-trans* photoisomerization is reduced by a competitive ESIPT channel in nonpolar solvent (cyclohexane). Upon specific solute–solvent interactions (methanol, acetonitrile) the intramolecular hydrogen bond in the *transoid-cis* form is perturbed, favoring the internal conversion S_1_ → S_0_ process as photostabilizing channel.

## Introduction

The proton transfer (PT) reaction is known to play an important role in a variety of biological and physical processes^1^. A proton may be transferred in either the ground or the excited electronic state along the intramolecular hydrogen bond of the molecule, or along the intermolecular hydrogen bond in its complexes^2,3^. PT plays many important functions in nature, e.g. the excited-state intramolecular proton transfer (ESIPT) process is known to be responsible for photostability of DNA bases due to the presence of multiple intermolecular hydrogen bonds linking the base pairs^4–6^. Moreover, the ESIPT process is a common reaction in many internally H-bonded organic molecules^7–10^ and thus may play an essential role in many applications, e.g., in organic photostabilizers^11–13^—the compounds used for protection of synthetic polymers against degradation caused by UV components of sunlight^14,15^. ESIPT can also lead to photochromic functionality, e.g. in Schiff bases^16^. The ESIPT phenomenon was utilized as a photoswitching mechanism in internally hydrogen-bonded molecules^17–19^. The search for proton cranes, in which a proton can be transferred over long distances through large-amplitude motion, is still a hot topic^20–22^.

The ESIPT process specifically between a hydroxyl group and an adjacent carbonyl group has been observed to proceed with a small energy barrier in quite a few chemical families. For example, rapid ESIPT has been implicated as a key factor in the photostability of naturally occurring hydroxyanthraquinone red colorants found in art masterpieces and illuminated manuscripts^23^. Ab initio studies on 5,6-dihydroxyindole (in which the 5-hydroxyl tautomerizes to a carbonyl) suggest a barrierless ESIPT as the key energy shunt pathway responsible for the photoprotective properties of eumelanin^24^. A similar process occurs in hydroxychromones^25,26^, hydroxyflavones^27^, and hydroxyquinolones^28^. Furthermore, ESIPT can either be coupled to, or compete with other processes. For example, 3-hydroxypicolinic acid engages in a double ESIPT^19,29^, and a designed quinoxalinylsalicylaldehyde can undergo either a normal ESIPT to the adjacent carbonyl, or an excited state long-range proton transfer (ESLRPT) to the more distant quinolinyl nitrogen, depending upon the excitation wavelength^30^.

**Figure 1:**
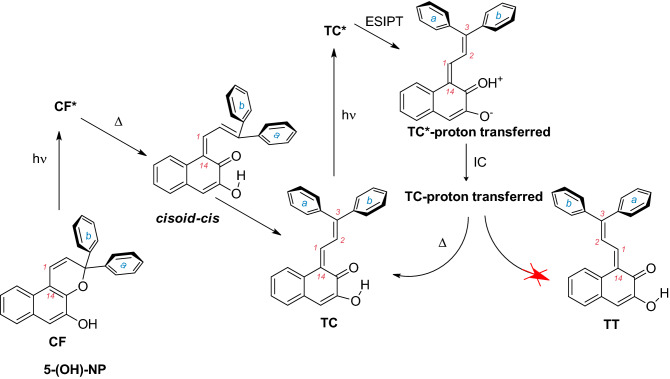
Expected photostabilization of the **TC** form due to the ESIPT process.

Importantly, the photophysics and photochemistry of molecular systems (especially their S_1_-state energetic landscape) can be tuned by chemical modifications that may activate the ESIPT pathway^18,29,31^, which upon strong intramolecular hydrogen bonding may proceed on subpicosecond time scale^32,33^ and with high efficiency at the expense of other competitive reaction channels in the excited state. For instance, such a strategy can be used to reduce triplet excited state formation in order to minimize side-effects in UV sunscreens (2-hydroxybenzophenone-based derivatives)^34^. Similarly, the present work leverages ESIPT to suppress the unwanted photochemical channel of *transoid-cis* → *transoid-trans* photoisomerization in 3*H*-naphthopyrans (**NP**). For this purpose, we chose 5-hydroxy-**NP** (**5-(OH)-NP**) as a model compound (Fig. 1).

*3H*-Naphthopyrans constitute a unique family of compounds that not only have advantageous photochromic properties for use in commercially available photochromic lenses, but also can be easily synthetically modified to obtain desired spectroscopic and photophysical properties^35–38^. The UV-induced coloration in **NP** compounds occurs via a barrierless photodissociative ring opening of the colorless pyranoid closed form (**CF**), leading to the formation of a colored open-form *transoid-cis* (**TC)** isomer^39^. During this process, or upon **TC** photoexcitation, a *transoid-trans* (**TT**) side product is produced if the excited-state energy landscape favours a *single-twist* rotation along the C_14_=C_1_ axis^40,41^. However, the presence of **TT** in photochromic systems may be unwanted in some applications, such as photochromic lenses, due to the substantially longer **TT** vs. **TC** lifetime (hours vs seconds), which leads to prolonged fading rates after the cessation of UV exposure^40,42^. The thermal fading **TT** → **TC** is hampered by a high energy barrier (ca. 1.3 eV^39^).

In this study we demonstrate with the 5-hydroxy-**NP** model compound that **TT** formation can be suppressed by the intervention of ESIPT. This principle may be important for the development of ESIPT-based **NP** derivatives for photochromic materials with reduced **TT** contribution.

## Experimental

### Materials

3,3-Diphenyl-5-hydroxy-3*H*-benzo[*f*]chromene (**5-(OH)-NP**) was synthesized following the procedures described in the Supplementary Information. In the time-resolved spectroscopic investigations cyclohexane, methanol and acetonitrile of spectroscopic grade from Sigma Aldrich were used for solution preparation. Studies were performed vs. a reference compound 3,3-diphenyl-3*H*-naphtho[2,1-*b*]pyran (**5-(H)-NP**) purchased from TCI.

### Time-resolved UV–Vis spectroscopy

Changes in UV–Vis absorption spectra and kinetics on a time scale of seconds were recorded for solutions in a 1 cm × 1 cm fused silica cuvette. Conditions of 21 °C were achieved by placing the sample into a temperature-controlled cuvette holder (Flash 300, Quantum Northwest) with stirring switched on. UV LED (λ_exc_ = 340 nm, Thorlabs M340L4) was used to induce the photochromic reaction. UV–Vis probing light was generated by a xenon lamp (Applied Photophysics), equipped with a bundle fiber. The probing beam was passed through a long-pass filter (WG 280) and an almost-closed iris to ensure low light intensity in the cell. The UV–Vis spectra were recorded by an Ocean Optics FLAME-T-VIS-NIR-ES USB spectrometer at the sampling rate of 20 spectra per second.

Femtosecond UV–Vis transient absorption spectra were obtained using a commercially available system (Ultrafast Systems, Helios)^43^. The ultrafast laser system consists of a short-pulse titanium-sapphire oscillator (Mai-Tai, Spectra Physics, 70 fs) followed by a high-energy titanium-sapphire regenerative amplifier (Spitfire Ace, Spectra Physics, 100 fs). The 800 nm beam was split into two beams to generate: (1) a pump (λ_exc_ = 444 nm) in the optical parametric amplifier (Topas Prime with a NirUVis frequency mixer) and (2) probe pulses—white light continuum in the UV–Vis (330–660 nm) range by using CaF_2_ plate. The remaining 800 nm photons in the probe pulse were filtered out before the sample: **5-(OH)-NP** solution in a quartz cell 2 mm thick with stirring. Photostationary state of **TC** population was obtained by the continuous UV LED irradiation at 340 nm. For selective excitation of the **TC** population, the excitation wavelength was set at λ_exc_ = 444 nm with a pump pulse energy of 1 μJ. The transient absorption data were corrected for chirp of white light continuum.

The obtained transient absorption spectra were analyzed using the global fitting procedure (ASUFIT program) and satisfactory fits were obtained with single- or double-exponential fits. Convolution with the instrument response function (200 fs, FWHM) was included in the fitting procedure. The accuracies of the obtained time-constants derived from analysis of transient absorption results were as follows: 5% (UV–Vis data in time window over tens/hundreds of seconds) and 10% (ultrafast UV–Vis data).

### Computational details

The equilibrium geometries of the **5-(OH)-NP** isomers in their closed-shell ground state (S_0_) were obtained with the MP2 method^44^ without imposing any symmetry constraints. The energy of the most stable form **CF**_**1**_ is the reference energy for higher energy structures. The excited-state (S_1_) equilibrium geometries were determined with the second-order algebraic diagrammatic construction ADC(2) method^45–47^. The correlation-consistent valence double zeta basis set with polarization functions on all atoms (cc-pVDZ)^48^ was used in these calculations, as well as in potential energy profiles and surfaces. The vertical excitation energies and response properties of the lowest excited singlet states were calculated using the CC2 methods^49,50^. The basis set augmented with the diffuse functions aug-cc-pVDZ was also used to compute vertical excitation energies of the molecular system. All calculations were performed using the TURBOMOLE program package^51^.

## Results and discussion

### Ground state energy landscape and vertical excitation energies of 5-(OH)-NP

The studied 5-hydroxy 3*H*-naphthopyran (**5-(OH)-NP**) possesses two closed-pyran **CF** minima in the ground electronic state (S_0_). The most stable is the **CF**_**1**_ form, which is stabilized by the O_5_–H$\cdots$O_4_ hydrogen-bond between the two neighboring oxygen atoms (Table 1). The second **CF**_**2**_ minimum, with the hydroxyl group rotated about 180°, is 0.156 eV higher in energy. As shown in Table 1, the S_0_ → S_2_ absorption maximum (strongest absorption) of the H-bonded **CF**_**1**_ form is bathochromic by 0.12 eV vs. the rotated **CF**_**2**_ form. Photoexcitation of the colorless **CF** form (**CF**_**1**_ or **CF**_**2**_) leads to the respective colored isomers (**TC**_**1**_ or **TC**_**2**_, respectively) in a barrierless process in the S_1_ excited state, the behaviour of which is similar to the previously studied unsubstituted parent **NP** molecule^39^. The photogenerated **TC**_**1**_ conformer is also stabilized by an intramolecular hydrogen bond *vs*. the rotated **TC**_**2**_ compartment, (0.407 eV vs. 0.752 eV). Such a large stabilization energy causes the **TC**_**1**_ form to predominate over the **TC**_**2**_ population under typical experimental conditions.

**Table 1:** Vertical excitation energy, *ΔE*^*VE*^ (in eV) and *λ*_*abs*_ (in nm), oscillator strength, *f*, and dipole moment, *μ*_*e*_ (in Debye), of the lowest excited singlet states calculated with the CC2/aug-cc-pVDZ method for the **5-(OH)-NP** ground state equilibrium forms optimized at the MP2/cc-pVDZ theory level. Dipole moment of the ground-state, *μ*_*g*_ (in Debye, MP2/cc-pVDZ).

S_0_ form		*ΔE*^*VE*^	*λ*_*abs*_	*f*	*μ*_*e*_
**Colorless CF forms**					
5-(OH)-CF (H-bonded), CF_1_	S_0_	0.00			***μ***_***g***_ = 2.3
	S_0_ → S_1_(ππ*)	3.81		0.035	1.00
	S_0_ → S_2_(ππ*)	3.88	**320**	**0.208**	2.74
	S_0_ → S_3_(nπ*)	4.71		0.009	2.32
	S_0_ → S_4_(ππ*)	4.75		0.058	3.35
	S_0_ → S_5_(nπ*)	4.88		0.009	6.26
5-(OH)-CF (rotated), CF_2_	S_0_	0.156^a^			***μ***_***g***_ = 1.1
	S_0_ → S_1_(ππ*)	3.81		0.036	1.55
	S_0_ → S_2_(ππ*)	4.00	**310**	**0.176**	3.87
	S_0_ → S_3_(nπ*)	4.69		0.000	3.44
	S_0_ → S_4_(nπ*)	4.81		0.003	8.26
**Colored TC forms**					
5-(OH)-TC (H-bonded), TC_1_	S_0_	0.407^a^			***μ***_***g***_ = 3.8
	S_0_ → S_1_(ππ*)	2.55	486	0.075	6.75
	S_0_ → S_2_(ππ*)	2.96	**420**	**0.771**	6.27
	S_0_ → S_3_(nπ*)	3.14		0.016	0.79
	S_0_ → S_4_(ππ*)	4.01		0.093	5.77
5-(OH)-TC (rotated), TC_2_	S_0_	0.752^a^			***μ***_***g***_ = 2.5
	S_0_ → S_1_(ππ*)	2.73	453	0.175	4.15
	S_0_ → S_2_(ππ*)	2.87	432	0.186	3.12
	S_0_ → S_3_(ππ*)	3.13	**396**	**0.509**	4.63
	S_0_ → S_4_(ππ*)	4.09		0.105	4.52
	S_0_ → S_5_(nπ*)	4.32		0.004	9.09
	S_0_ → S_6_(ππ*)	4.40		0.205	3.17
**Colored TT forms**					
5-(OH)-TT (H-bonded), TT_1_		0.438^a^			***μ***_***g***_ = 3.7
	S_0_ → S_1_(ππ*)	2.68	463	0.122	5.82
	S_0_ → S_2_(ππ*)	3.06	**405**	**0.670**	7.42
	S_0_ → S_3_(ππ*)	3.33		0.023	1.08
	S_0_ → S_4_(ππ*)	4.11		0.062	5.72
	S_0_ → S_5_(ππ*)	4.37		0.216	2.89
	S_0_ → S_6_(ππ*)	4.39		0.022	11.96
5-(OH)-TT (rotated), TT_2_		0.760^a^			***μ***_***g***_ = 2.8
	S_0_ → S_1_(ππ*)	2.82	**440**	**0.206**	3.92
	S_0_ → S_2_(ππ*)	3.11	**399**	**0.308**	1.40
	S_0_ → S_3_(ππ*)	3.25	**381**	**0.284**	5.48
	S_0_ → S_4_(ππ*)	4.20		0.074	4.55
	S_0_ → S_5_(ππ*)	4.37		0.178	4.88
	S_0_ → S_6_(ππ*)	4.46		0.028	8.22

^a^Difference in adiabatic energy (E^a^, in eV) relative to the closed form, **CF**_**1**_, calculated at the MP2/cc-pVDZ theory level.

Significant values are in [bold].

The calculated S_0_-state energy barrier for free rotation of the hydroxyl group (**TC**_**2**_ → **TC**_**1**_) is + 0.17 eV or + 0.18 eV, depending on the rotation side (MP2/cc-pVDZ). Again, similar to **CF**, the absorption maximum of H-bonded **TC**_**1**_ is slightly red-shifted *vs*. the rotated **TC**_**2**_ isomer (see Table 1). Stabilization of **TC**_**1**_ by intramolecular hydrogen bonding explains why for freshly prepared solutions of **5-(OH)-NP** a small amount of **TC** species is observed by a weak absorption band with maximum at 443 nm (Fig. S1). Its content is roughly estimated at 0.8% in cyclohexane, and a similar amount of 1.0–1.5% was estimated in CDCl_3_ by ^1^H-NMR spectra integration (see Supplementary Information). Interestingly, the **TC** band absorption is less pronounced in methanol and acetonitrile (Fig. S1), likely due to disruption of the internal hydrogen bond in **TC** by solute–solvent specific interactions. Such an equilibrium between **TC** and **CF** has been reported for a similar **NP** derivative^52^. To explore this phenomenon, various **TC** and methanol (1:1) complexes were calculated (Fig. S2A). The most stable complex is **MC1** with one hydrogen bond formed between the O_5_H hydroxyl group and the methanol acting as a proton acceptor, and a second H-bond formed between the O_4_ oxygen atom and the methanol acting as a proton donor. Such double hydrogen bond formation stabilizes the **TC–methanol** complex. However, the intramolecular hydrogen bond is weakened (length is 2.373 Å, Fig. S2A) vs the isolated **TC** molecule in the gas phase (1.947 Å). In the case of complexes with acetonitrile (Fig. S2B), interactions are related to ππ stacking interactions and hydrogen bonding.

### Thermally activated fading process of the TC isomer to colorless CF

**Figure 2:**
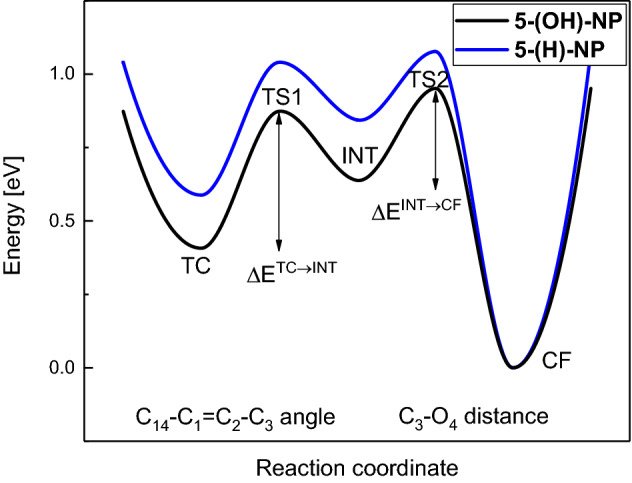
Thermal decoloration of **TC** → **CF** for **5-(OH)-NP** compound (black line) involves two reaction steps **TC** → **INT** and **INT** → **CF** (the first step is reversible). S_0_-state potential-energy profiles were computed with aid of the MP2/cc-pVDZ method. The reference compound **5-(H)-NP** (blue curve) is traced according to reported data^53^.

The **TC** isomer plays a key role as a coloured species in the photochromic reaction of **NP** derivatives. **TC** decay occurs in a two-step reaction leading to **CF** via a high-in-energy c*isoid-cis* intermediate **INT** form. The timescale of the **TC** form depopulation depends strongly on the ratio of the S_0_ state energy barriers, ΔE, separating this *cisoid-cis* intermediate from the neighboring stable conformers: colored **TC** (ΔE^INT-TC^) and colorless **CF** (ΔE^INT-CF^), see Fig. 2^41,53^.

The **TC**_**1**_ relative energy of 0.407 eV for **5-(OH)-NP** is much lower than that for the **5-(H)-NP** derivative (0.588 eV)^39^, mainly due to the internal hydrogen bond in the **TC**_**1**_ form of **5-(OH)-NP**. Conversely, the larger relative energy of **TC**_**2**_ (0.752 eV) is due to O_4_$\cdots$O_5_ repulsion, which is absent in **5-(H)-NP**. The relative energy of the rotated **TC**_**2**_ isomer is higher (0.752 eV) than that of **TC**_**1**_ (0.407 eV); consequently, in inert solution such as cyclohexane the equilibrium is shifted towards **TC**_**1**_.

The **TC** isomers constitute relatively deep minima on the S_0_-state minimum potential-energy profile, separated from the **INT** intermediate by the ΔE^TC-out^ energy barrier of + 0.466 eV in the case of H-bonded **TC**_**1**_ (Table 2), which is slightly larger than for **5-(H)NP** (+ 0.452 eV)^39^. Despite the large barrier, the process leading to **CF** is thermally available due to its exothermic character. However, the high-in-energy intermediate **INT** must first be populated. Once **INT** is formed, it turns out that the energy barrier (ΔE^INT-CF1^) for the forward process (**INT**_**1**_ → **CF**_**1**_) is much larger (+ 0.313 eV) than the corresponding barrier (ΔE^INT-TC1^) toward T**C**_**1**_ (+ 0.235 eV). Thus, one can expect recovery of the **TC**_**1**_ isomer as the most probable path (Fig. 2), which suggests a rationale for the long apparent **TC**_**1**_ lifetime in solution for **5-(OH)-NP** vs. **5-(H)-NP**.

**Table 2:** Energetics of the two-step process of the **TC** form depopulation (**TC** ↔ **INT** → **CF**) for **5-(OH)-NP** vs. **5-(H)-NP**. Adiabatic S_0_-state energies, *E*^*a*^, in eV, and dipole moment, *μ*_*g*_, in Debye, for the relevant minima: **TC**, **INT**, **CF**, and transition states separating these minima: **TS1** and **TS2**, calculated at the MP2/cc-pVDZ theory level. Results for **5-(H)-NP** are from reference^53^.

Form	**TC** E^a^/eV μ_g_/D	∆E^TC-out^	**TS2** E^a^/eV μ_g_/D	∆E^INT-TC^	**INT** E^a^/eV μ_g_/D	∆E^INT-CF^	**TS1** E^a^/eV μ_g_/D	**CF** E^a^/eV μ_g_/D
								
5-(OH)-NP (H-bonded)	0.407 3.8	+ 0.466	0.873 2.0	+ 0.235	0.638 2.3	+ 0.313	0.951 2.9	0.00 2.60
								
5-(OH)-NP (rotated)	0.752 2.5	+ 0.431	1.183 2.7	+ 0.197	0.986 1.5	+ 0.246	1.232 0.3	0.156 1.1
								
5-(OH)-NP with methanol	0.555 4.9 **MC1**	+ 0.437	0.992 2.6	+ 0.252	0.740 3.0	+ 0.281	1.021 3.7	0.000 3.8
								
5-(H)-NP	0.588 2.8	+ 0.452	1.040 2.9	+ 0.196	0.844 1.5	+ 0.233	1.077 0.7	0.000 0.9

### Apparent TC fading rate and lack of TT population in changes of UV–Vis absorption spectra

**Figure 3:**
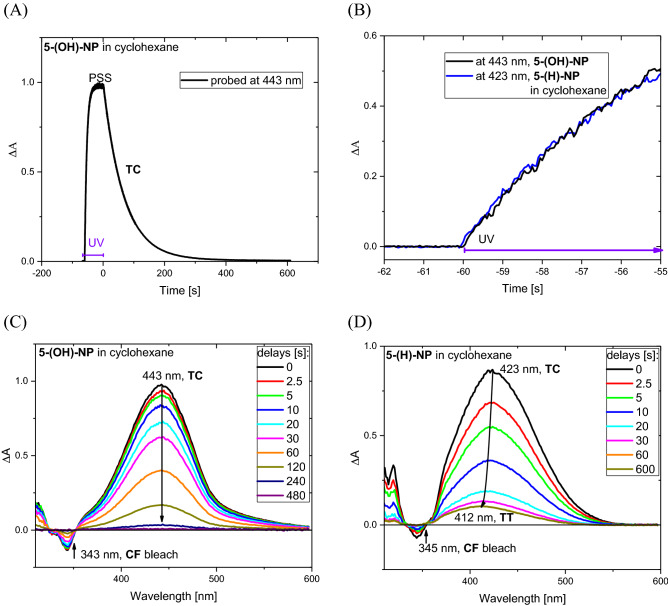
(**A**) Changes in absorption at probe 443 nm caused by UV irradiation. (λ = 340 nm, 4 mW/cm^2^) of **5-(OH)-NP** in cyclohexane at 21 °C. (**B**) The initial rise in absorption upon switching on UV irradiation for two solutions **5-(OH)-NP** and **5-(H)-NP** in cyclohexane prepared with the same absorption (A(340 nm, 1 cm) = 0.32). (**C**, **D**) Evolution of ΔA spectra after ceasing UV irradiation at t = 0 s for **5-(OH)-NP** and **5-(H)-NP**, respectively.

In our previous studies of **NP** photochromic reactions, LED light at λ = 365 nm was used for photoexcitation^39–41^. However, since the **5-(OH)-NP** absorption spectrum is blue-shifted in comparison to **5-(H)-NP** (see Fig. S1), LED light at λ = 340 nm was selected for this investigation. UV irradiation of **5-(OH)-NP** in cyclohexane for 60 s produces a photostationary state (PSS, Fig. 3A).

Figure 3B shows that the initial rise of the **TC** absorption signal is very similar to **5-(H)-NP**, so one can estimate a similar quantum yield for **TC** formation in cyclohexane (0.745^39^). Figure 3C presents the evolution of absorption for **5-(OH)-NP** after ceasing UV irradiation at the moment t = 0 s. The initial absorption spectrum shows a positive **TC** absorption band with a maximum at 443 nm, and a negative band at 343 nm due to **CF** bleach. Subsequently, the decay of the **TC** band occurs more slowly in **5-(OH)-NP** than for **5-(H)-NP** (Fig. 3C *vs.* 3D), with apparent **TC** lifetimes at 21 °C of 66 s and 9.2 s, respectively, according to global analysis (see Fig. S3).

Note that this **TC** lifetime elongation effect was predicted by theoretical calculations (section above). Upon decay of the **TC** absorption band, a practically baseline level is reached after a few hundreds of seconds (Fig. 3C), in contrast to **5-(H)-NP** (Fig. 3D), which shows the presence of the **TT** absorption band. This demonstrates that insertion of the hydroxyl group at position 5 suppresses the **TT** formation channel. A substantial **TT** reduction is clearly observed (compare Fig. 3C *vs.* 3D), and only a very low concentration of **TT** can be detected for **5-(OH)-NP** (see Fig. S3). The reduced yield of the photoisomerization path **TC** → **TT** could be explained by a strong competitive channel, which is probably ESIPT in cyclohexane. As we mentioned above, the intramolecular hydrogen bond in **TC** (from **5-(OH)-NP**) is likely disrupted in methanol or acetonitrile solution, so the ESIPT process is less probable in these solvents. Nevertheless, reduced **TT** formation is observed (see Figs. S4 and S5). The photostabilization of the **TC** species in these solvents is probably mediated by solvent polarity^54^—note that calculated dipole moment for **TC** in the S_1_ state is high (Table 1). Stabilization of **TC** in the S_1_ state in polar solvent may facilitate conical intersection CI (S_1_/S_0_) leading to the S_0_ state with a less advanced change in the twist angle C_14_=C_1_, thus **TT** formation is disfavored. This trend, although less pronounced, has been already observed for the parent compound **5-(H)-NP** in acetonitrile^54^.

According to Table 3, the apparent S_0_ ground state **TC** lifetime is longer for **5-(OH)-NP** vs. **5-(H)-NP**. The largest difference is observed in cyclohexane (factor of 7) due to the presence of the intramolecular hydrogen bond for **5-(OH)-NP** stabilizing the **TC** form. The presumed breaking of the intramolecular H-bond in polar solvents such as methanol and acetonitrile has been also postulated for structurally related 3-hydroxychromone^26,55^. Even if a weakened intramolecular hydrogen bond in **TC** is present in methanol (Fig. S2), calculations performed for **MC1** complex show a decrease in the ΔE^INT-CF^ barrier and an increase in ΔE^INT-TC^ barrier in comparison to the isolated **5-(OH)-NP **(H-bonded) molecule (see Table 2). Thus, the shortening of the S_0_ **TC** lifetime in methanol in relation to cyclohexane can be rationalized.

**Table 3:** Photophysical properties of **TC** in solution: S_0_ absorption band maximum and lifetime τ_S0_ with 5% accuracy (at 21 °C).

Compound	Solvent	S_0_(TC) λ_abs_^max^, nm	τ_S0_, s
5-(OH)-NP	Cyclohexane	443	66
	Acetonitrile	443	27.5
	Methanol	437	50
5-(H)-NP	Cyclohexane	423	9.2
	Acetonitrile	427	8.5
	Methanol	434	12

### Properties of the excited TC isomer

**Figure 4:**
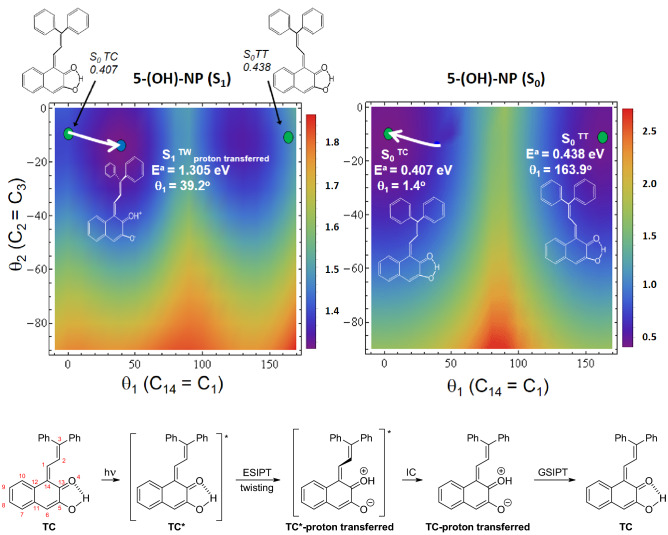
Minimum potential-energy surface of the lowest excited (left) and the ground electronic state (right) of the **5-(OH)-TC** (H-bonded) species mimicking system evolution, plotted as a function of θ_1_(C_14_=C_1_) and θ_2_(C_2_=C_3_) torsional angle coordinates. Green circles represent the Franck–Condon regions of the ground-state **S**_**0**_^**TC**^ and **S**_**0**_^**TT**^ local minima. Blue circle represents **S**_**1**_^**TC**^ minimum populated after **S**_**0**_^**TC**^ photoexcitation. White arrows show the evolution of the system. The results were obtained with the aid of the ADC(2)/cc-pVDZ method, for the excited state (S_1_), and with the MP2/cc-pVDZ, for the ground state (S_0_). The reaction scheme below shows steps in the photocycle free from **TT** generation.

For most **NP** derivatives photoexcitation of the **TC** isomer to its Franck–Condon region results in an excited S_1_-state geometrical relaxation, in which rotations about the two double bonds C_14_=C_1_ and C_2_=C_3_ are activated^40,41^. But in **5-(OH)-TC** the S_1_ state relaxation is additionally accompanied by the barrierless excited-state intramolecular proton transfer (ESIPT) from O_5_ to O_4_ to produce a proton-transferred species. The minimum energy of the TC*-proton transferred species of **5-(OH)-NP** was found to be lower in energy (E^a^ = 1.305 eV) *vs*. the corresponding TC* species from **5-(H)-NP** (E^a^ = 1.948 eV)^40^, due to presence of the stabilizing intramolecular hydrogen bond O_5_$\cdots$H-O_4_ (see Fig. 4). Another observation is that the relaxed S_1_ **TC** minimum occurs with an optimized θ_1_ (C_14_=C_1_) dihedral angle of ~ 39° (see left square in Fig. 4), much lower than 90°, where the S_0_-state energy barrier is seen (right square in Fig. 4). Thus, upon S_1_ → S_0_ internal conversion, a species in the S_0_ state is produced with geometry which, in the next step, follows the S_0_-state gradient toward the S_0_ **TC** global minimum. This barrierless process confirmed by geometry optimization is accompanied by the back proton transfer from O_4_ to the O_5_ oxygen atom (i.e. ground state intramolecular proton transfer, GSIPT). Note that alternative pathways, such as **TC** → **TT** isomerization, are suppressed due to a S_0_-state high-energy wall (Fig. 4). Moreover, note that the θ_1_(C_14_=C_1_) dihedral angle of 39° found for the **TC** S_1_-state minimum (see Table S1) is one of the smallest θ_1_ values for derivatives we have studied^40,41^, which makes the **5-(OH)-NP** molecule well situated for suppression of **TT** formation.

### Confirmation of ESIPT by ultrafast transient absorption studies

**Figure 5:**
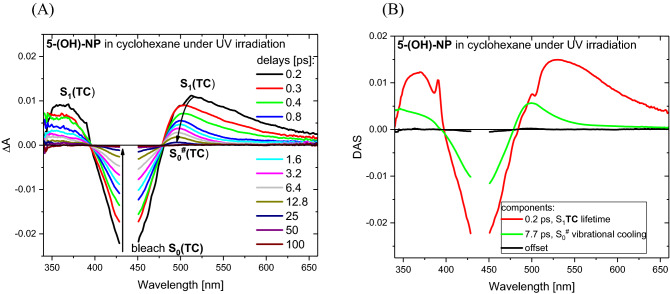
(**A**) UV–Vis transient absorption spectra recorded for **TC** solution in cyclohexane upon the laser pulse excitation at λ = 444 nm. PSS with a constant **TC** population was achieved by a continuous irradiation of **5-(OH)-NP** cyclohexane solution with LED at λ = 340 nm. (**B**) Decay associated spectra obtained with global analysis using bi-exponential function.

To confirm the ESIPT process predicted by theory in the gas phase, for experimental work the inert solvent cyclohexane was selected. Thus, for ultrafast studies, a solution of **5-(OH)-NP** in cyclohexane was prepared and the sample was continuously irradiated by LED at 340 nm to generate a photostationary state (PSS). As described above, in PSS practically the only colored form is **TC**. In the pump-probe experiment, a laser-pulse excitation wavelength of 444 nm was chosen for selective **TC** photoexcitation. Figure 5A shows UV–Vis transient absorption recorded in the time window from 0.2 to 100 ps.

At the initial delay of 0.2 ps the positive bands at 360 and 520 nm are clearly observed. Two assignments for these bands can be considered. One is that the initial positive spectrum belongs to the population product—TC*-proton transferred, since the transfer of the proton can be faster than our temporal resolution (< 200 fs), in agreement with theoretical calculations (barrierless process) and the ultrafast character of such processes in analogous molecular systems (3-hydroxyflavone^27^). But such an assignment does not agree with the calculated electronic transition S_1_ → S_n_ for TC*-proton transferred (see Table S2). The alternative assignment for the initial positive band is TC*. Indeed, the spectral shape is reminiscent of the recorded data for TC* from **5-(H)-NP**^54^. Thus, the positive bands at 360 and 520 nm correspond to S_1_ → S_n_ and S_1_ → S_m_ transitions of TC* population (n > m), while the negative band peaking at about 440 nm can be attributed to **TC** bleaching. On the basis of global analysis (Fig. 5B), the **TC** excited-state lifetime is only 0.2 ps vs. 0.8 ps (**5-(OH)-NP** vs. **5-(H)-NP**). This shortened **TC** lifetime effect can be explained by ESIPT, which drives the system toward the conical intersection CI(S_1_/S_0_) region much faster. The proton-transferred **TC** excited state is not observed in the experiment due to a low instantaneous concentration. The back proton transfer occurring in the S_0_ state is barrierless according to calculations, so that it also probably has ultrafast character^56^. Thus, the evolution of transient spectra 0.8–100 ps (Fig. 5A) reflects vibrational cooling of the nascent hot **TC** species in the electronic ground state S_0_^#^. The spectrally wide S_0_^#^ **TC** absorption band in comparison to the vibrationally relaxed S_0_ **TC** absorption band is an expected feature^57^. The time-constant of vibrational cooling is 7.7 ps obtained from global fitting (Fig. 5B).

### TC excited state deactivation in polar and hydrogen-bonding solvents

Upon changing from cyclohexane to methanol solution, the recorded transient absorption spectra are similar (Fig. S6), but the population of **TC** in the singlet excited state shows a longer lifetime (0.8 ps in methanol *vs.* 0.2 ps in cyclohexane, Table S3), suggesting ESIPT in that solvent, if present, is a minor deactivation path. Moreover, one would expect a slower ESIPT for **5-(OD)-NP** in MeOD than for **5-(OH)-NP** in MeOH, due to the difference in mass between deuterium and hydrogen, as reported in other molecular systems^58,59^. However nearly identical transient absorption spectra and kinetics were collected for **5-(OD)-NP** (in MeOD) and **5-(OH)-NP** (in MeOH), which supports the hypothesis of minor ESIPT involvement in these solvents due to a high competition from internal conversion S_1_ → S_0_ caused by a change in the **TC** S_1_ geometry in the C_14_=C_1_–C_2_=C_3_ bridge, by analogy with **5-(H)-NP**^39,54^. In the polar acetonitrile, the transient absorption spectra for **TC** are similar to those in cyclohexane and methanol. The **TC** S_1_ state lifetime is 0.5 ps in acetonitrile (Fig. S7), thus longer than in cyclohexane (0.2 ps), but shorter in relation to methanol (0.8 ps, Table S3). To rationalize the decrease of τ_S1_ in acetonitrile *vs.* methanol, we note that the **TC** S_1_ state dipole moment is substantially high (Table 1). Thus, one expects that the high acetonitrile polarity exerts a stabilizing effect for **TC** in the excited state and S_1_ → S_0_ internal conversion is facilitated. In methanol, on the other hand, strong solute–solvent specific interactions can slow down the geometry evolution needed for reaching the conical intersection, responsible for S_1_ → S_0_ internal conversion. Interestingly, in acetonitrile and methanol τ_S1_ TC* lifetimes are longer for **5-(OH)-NP** than **5-(H)-NP** (Table S3). The presence of the hydroxy group strengthens solute–solvent interactions, thereby hampering the change in TC* geometry.

## Conclusions

Calculations in the gas phase show that the ESIPT process predominates in **TC** S_1_ state deactivation over the **TC** → **TT** photoisomerization, hindering formation of the **TT** isomer. Internal conversion (S_1_ → S_0_) may take place during the ESIPT process before the double-bond rotations are activated in the excited state. The ESIPT process is experimentally observed in cyclohexane by a shortening of the singlet excited **TC** lifetime to 0.2 ps in relation the unsubstituted compound (0.8 ps for **5-(H)-NP**^54^). Such fast dynamics of ESIPT has been reported in structurally related 3-hydroxychromone^26^. In other solvents, such as methanol or acetonitrile, ESIPT plays a less important role in **TC** excited state deactivation, since the intramolecular hydrogen bond is disrupted by solute–solvent specific interaction. The polar **TC** S_1_ species is stabilized by solvent polarity, and the **TC** S_1_ deactivation mainly occurs through the internal conversion process S_1_ → S_0_ induced by a change in geometry upon activation of the two double bonds present in the C_14_=C_1_–C_2_=C_3_ bridge. Stabilization of **TC** S_1_ species in polar solvent lowers the S_1_-state potential-energy profile (vs. S_0_-state), thus the conical intersection CI(S_1_/S_0_) produces the S_0_ state with less advanced change in the C_14_=C_1_ twist angle. Consequently, **TT** formation is disfavoured. On the other hand, different types of solvent-molecule interactions (dipole–dipole, π–π stacking and hydrogen bonding) slow down the TC* geometry evolution towards the conical intersection CI (S_1_/S_0_).

These main pathways (ESIPT or polarity-mediated IC) appear to reduce photoisomerization via the channel **TC** → **TT**. This explains the reduction of the **TT** population in the photochromic reaction using continuous UV irradiation performed for **5-(OH)-NP** in comparison to the parent compound **5-(H)-NP**.

An unintended consequence of 5-hydroxy substitution is a weak residual color in **5-(OH)-NP** solutions due to the presence of small quantities (ca. 1%) of the colored **TC** isomer produced through thermal equilibrium in cyclohexane solution. This is a result of the intramolecular hydrogen bond present in the structure, stabilizing its relative energy *vs*. the closed colorless form **CF**. Breaking the intramolecular hydrogen bond in methanol or acetonitrile by solute–solvent specific interactions shifts the equilibrium towards **CF**.

## Supplementary Information

Supplementary Information.

## Data Availability

The datasets generated during the current study are available from the corresponding author on request.
