# Supplementary material for: Blockade of persistent colored isomer formation in photochromic 3H-naphthopyrans by excited-state intramolecular proton transfer
Source: Sci Rep. 2022 Nov 10;12:19159. doi: 10.1038/s41598-022-23759-9 (PMC9649631; doi:10.1038/s41598-022-23759-9)
Supplement: Supplementary file 1 — Supplementary Information. [file 41598_2022_23759_MOESM1_ESM.pdf]

Supplementary data for

Blockade of persistent colored isomer formation in photochromic 3*H*-naphthopyrans by excited-state intramolecular proton transfer

Błażej Gierczyk[a], S. Shaun Murphree[b], Michał F. Rode[c,*] and Gotard Burdzinski[d,*]

[a] Faculty of Chemistry, Adam Mickiewicz University in Poznań, Uniwersytetu Poznańskiego 8, 61-614 Poznań, Poland,

[b] Department of Chemistry, Allegheny College, 520 North Main Street, Meadville, PA, USA

[c] Institute of Physics, Polish Academy of Sciences, Aleja Lotników 32/46, 02-668 Warsaw, Poland, E–Mail: mrode@ifpan.edu.pl (M.R.),

[d] Faculty of Physics, Adam Mickiewicz University in Poznań, Uniwersytetu Poznańskiego 2, 61-614 Poznań, Poland, E–Mail: gotardb@amu.edu.pl (G.B.)

Synthesis of compound 3,3-diphenyl-5-hydroxy-3*H*-benzo[*f*]chromene **2**

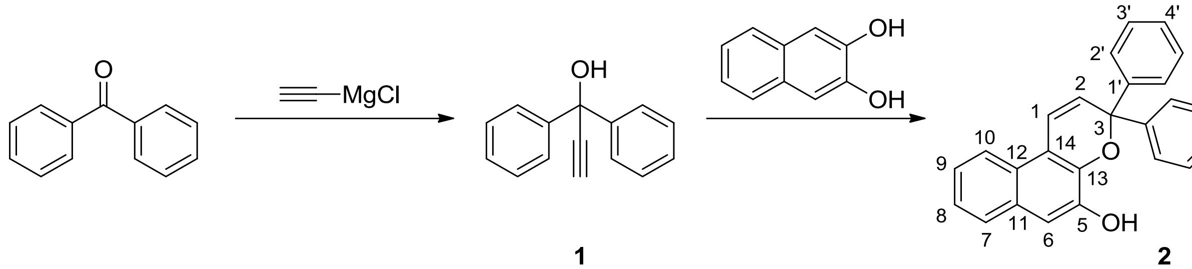

1 **2**

1,1-Diphenyl-2-propyn-1-ol (1)

Ethynylmagnesium chloride (100 mL, 0.5 M solution in THF, 50 mmol) was added dropwise to a solution of benzophenone (5 g, 27.4 mmol) in dry THF (100 mL) at room temperature. The mixture obtained was stirred for 24 h, and then the reaction was quenched by adding NH_4Cl (200 mL, 5% aqueous). The product was extracted with diethyl ether (2 x 100 mL), the organic layer was dried over anhydrous Na_2SO_4, and the solvent was evaporated. The product obtained as a brownish oil (4.73 g; 83%) was used without further purification.

^{1}H NMR ($CDCl_3$): δ 7.63 (*pseudo*d, 2H); 7.34 (*pseudo*t, 2H); 7.28 (*pseudo*t, 1H); 3.39 (s, 1H); 2.87 (s, 1H).

^{13}C NMR ($CDCl_3$): δ 144.5; 128.2; 127.7; 125.9; 86.5; 75.3; 74.1.

3,3-Diphenyl-5-hydroxy-3*H*-benzo[*f*]chromene (2)

A solution of 1,1-diphenyl-2-propyn-1-ol (1.00 g, 4.8 mmol), 2-naphthol (0.69 g, 4.8 mmol), triethyl orthoformate (2 mL, 12 mmol), and pyridine tosylate (50 mg, catalytic) in dry 1,2-dichloroethane (50 mL) was heated over 5 h under reflux in an inert atmosphere and protected from light. After cooling, the solvent was evaporated in vacuo. The residue was dissolved in dichloromethane (25 mL), and this solution was washed with water (2 x 20 mL). The organic layer was dried over Na_2SO_4, evaporated, and the product obtained was purified by column chromatography on SiO_2 using chloroform/hexane 1:1 (v/v) as eluent. The product was dissolved in a small amount of hot dichloromethane and diluted with methanol. After evaporation of the dichloromethane, orange crystals of **2** were collected by filtration; yield 1.21 g (72%; m.p. 161-163°C).

^{1}H NMR ($CDCl_3$): 7.89 (d, 1H, 8.4Hz; H7); 7.62 (d, 1H, 8.1 Hz; H10); 7.44 (*pseudo*d, 4H; H2'); 7.35 (m, 1H; H8); 7.34 (*pseudo*t, 4H; H-3'); 7.33 (d, 1H, 10.0 Hz; H1); 7.31 (m, 1H; H9); 7.30 (*pseudo*t, 2H; H-4'); 7.22 (s, 1H; H6); 6.25 (d, 1H, 10.0 Hz; H2); 5.90 (s, 1H; O**H**).

^{13}C NMR ($CDCl_3$): δ 144.3 (C5); 143.9 (C1'); 139.8 (C13); 129.8 (C11); 128.3 (C3'); 128.0 (C4'); 127.8 (C2); 127.2 (C10); 127.0 (C2'); 124.7 (C12); 124.4 (C9); 124.2 (C8); 121.2 (C7); 119.5 (C1); 114.4 (C14); 110.8 (C6); 83.9 (C3).

The ^{1}H NMR spectrum of **2** shows the presence of open structure (**TC-2**) in equilibrium with the chromene tautomeric form. The presence of **TC-2** is manifested by the characteristic signals at 8.81 ppm (d, 11.9 Hz; H2) and 7.88 ppm (d, 11.9 Hz; H1). Its content is 1.0-1.5% based on ^{1}H NMR spectra integration.

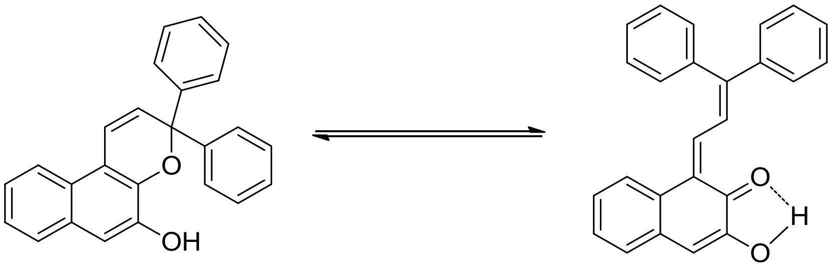

2 **TC-2**

1,1-Diphenyl-2-propyn-1-ol (**1**) in $CDCl_3$

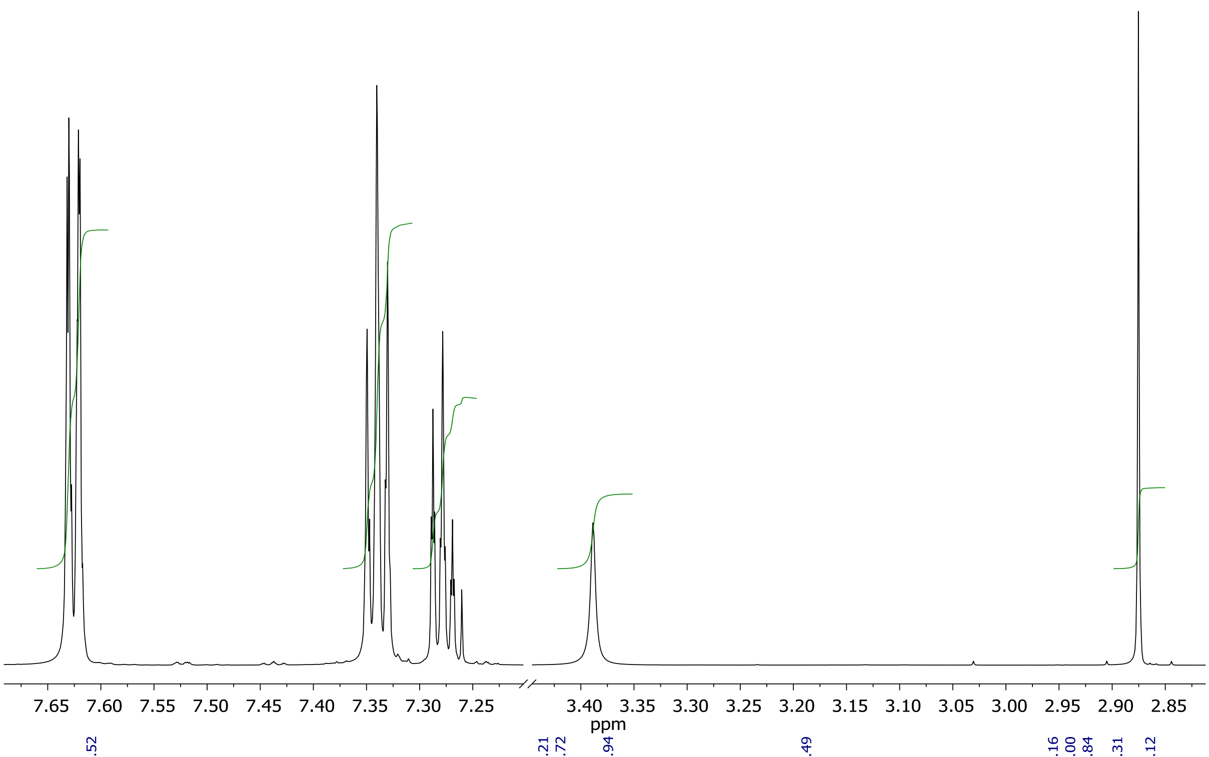

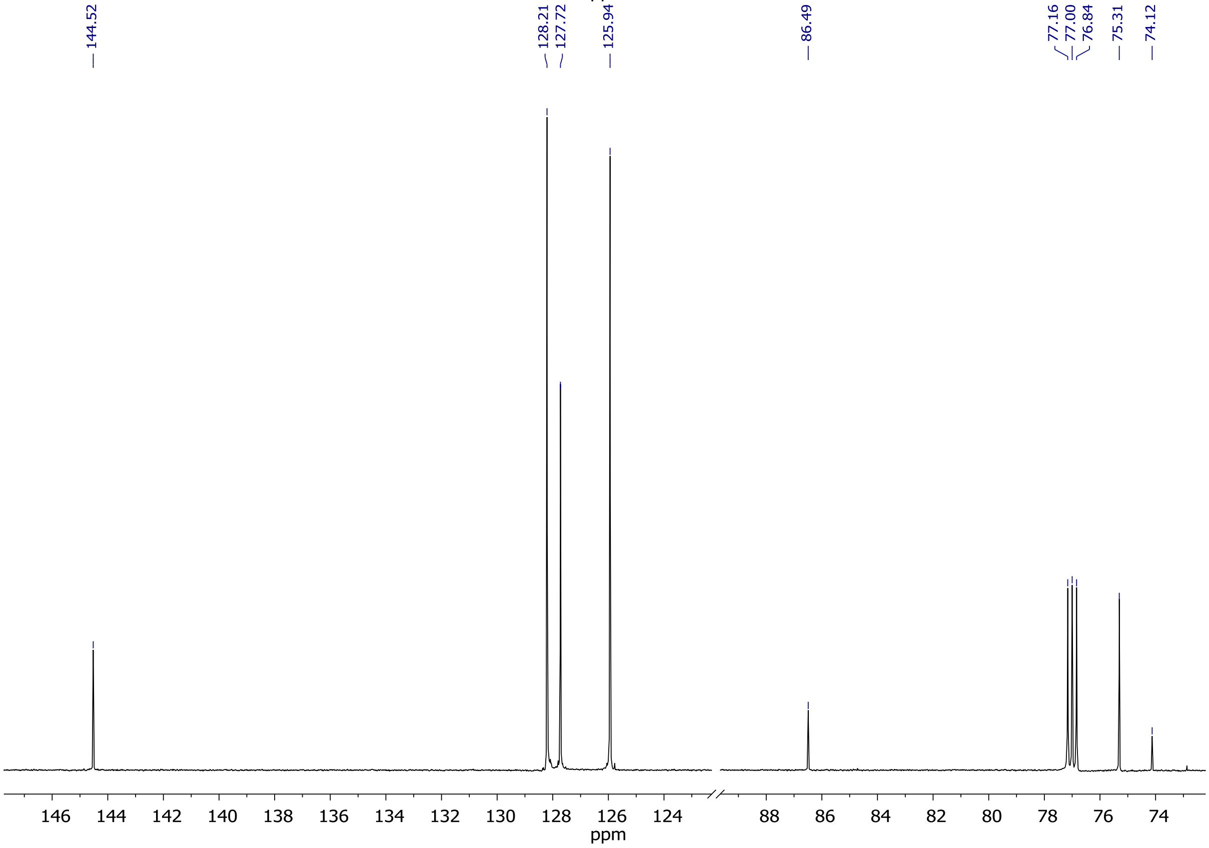

3,3-Diphenyl-5-hydroxy-3*H*-benzo[*f*]chromene (**2**) in $CDCl_3$

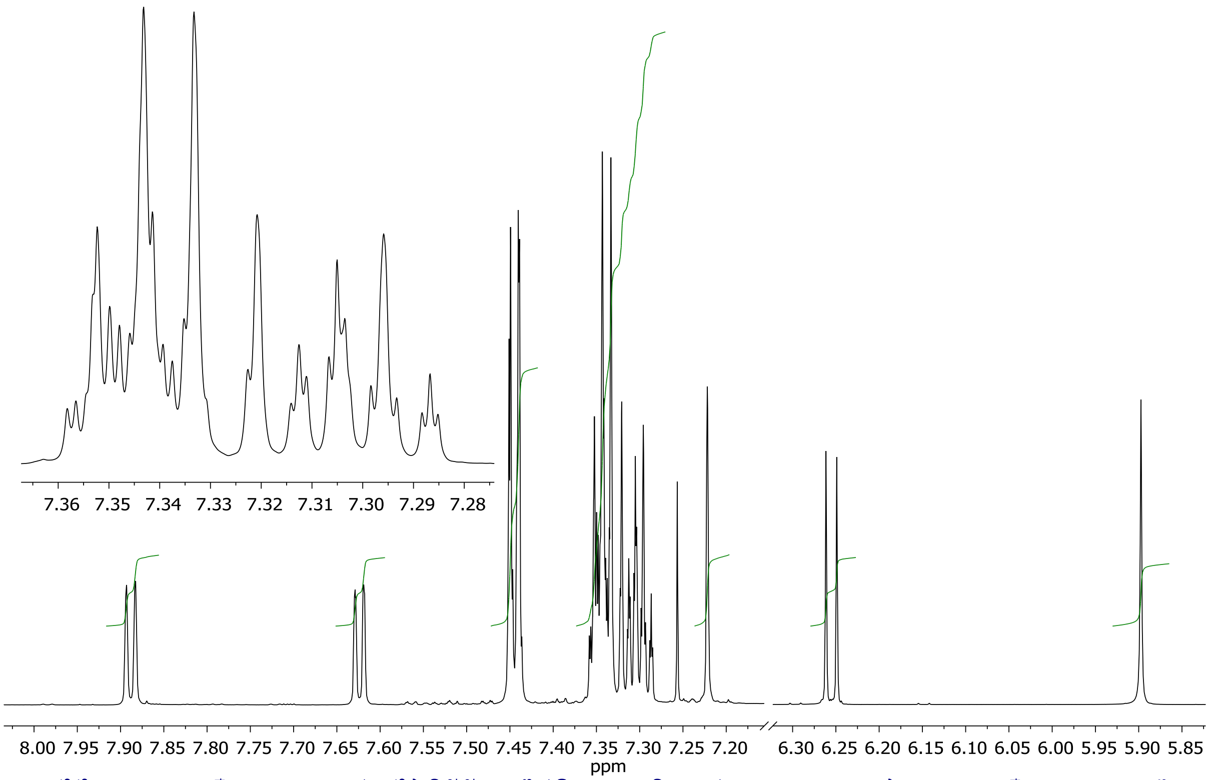

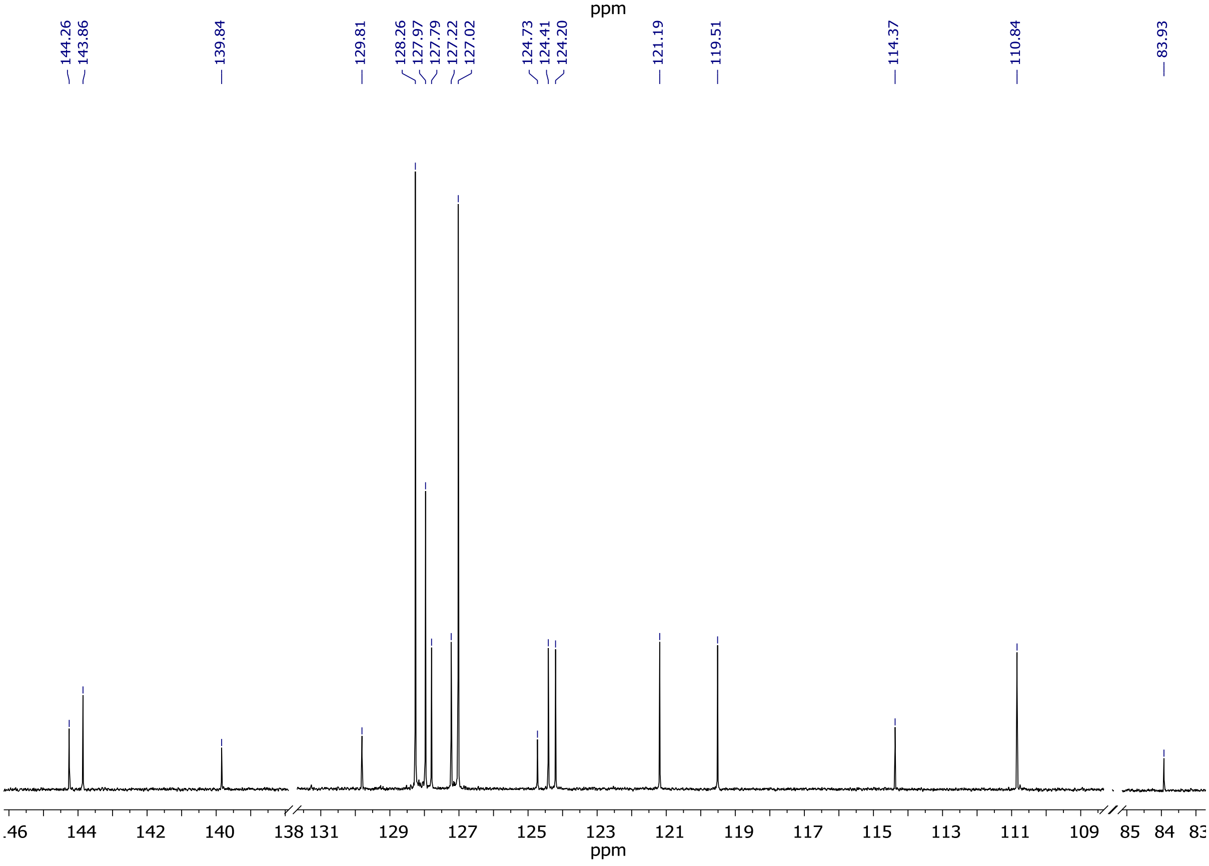

Low-field region of the ^{1}H-^{1}H COSY of 3,3-diphenyl-5-hydroxy-3*H*-benzo[*f*]chromene (**2**) – H1 and H2 signals of open tautomer (TC-2).

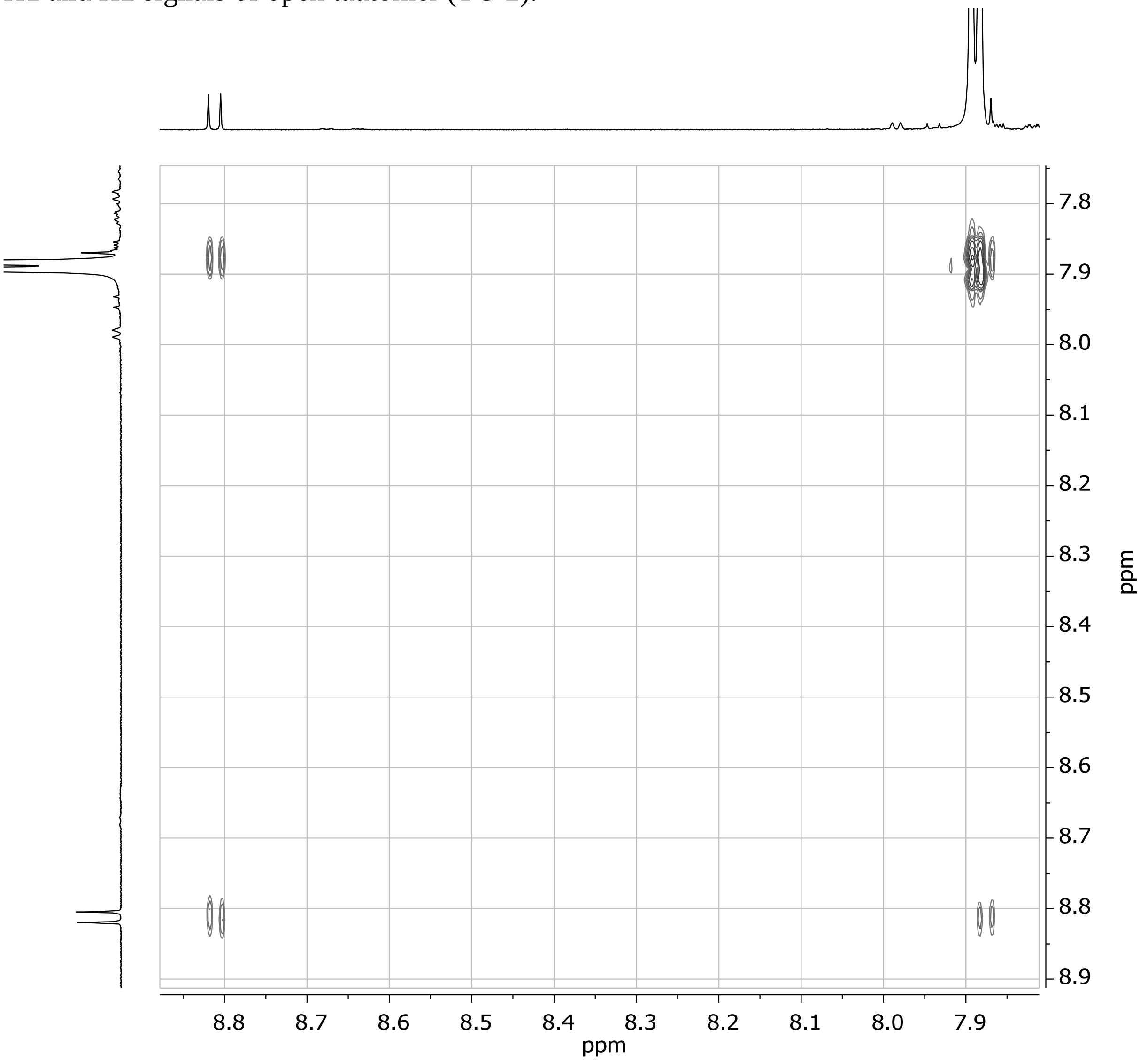

Figure S1. Stationary UV-vis spectra for **5-(OH)-NP** and **5-(H)-NP** in cyclohexane shown in spectral ranges: 200–400 nm (A) and 350–500 nm (B). Note that the **5-(OH)-NP** solution shows a weak absorption band with a maximum at 443 nm caused by residual **TC** population in thermal equilibrium **CF**↔**TC**. This feature is absent in other solvents, such as methanol and acetonitrile (C), and for the reference compound **5-(H)-NP** (D).

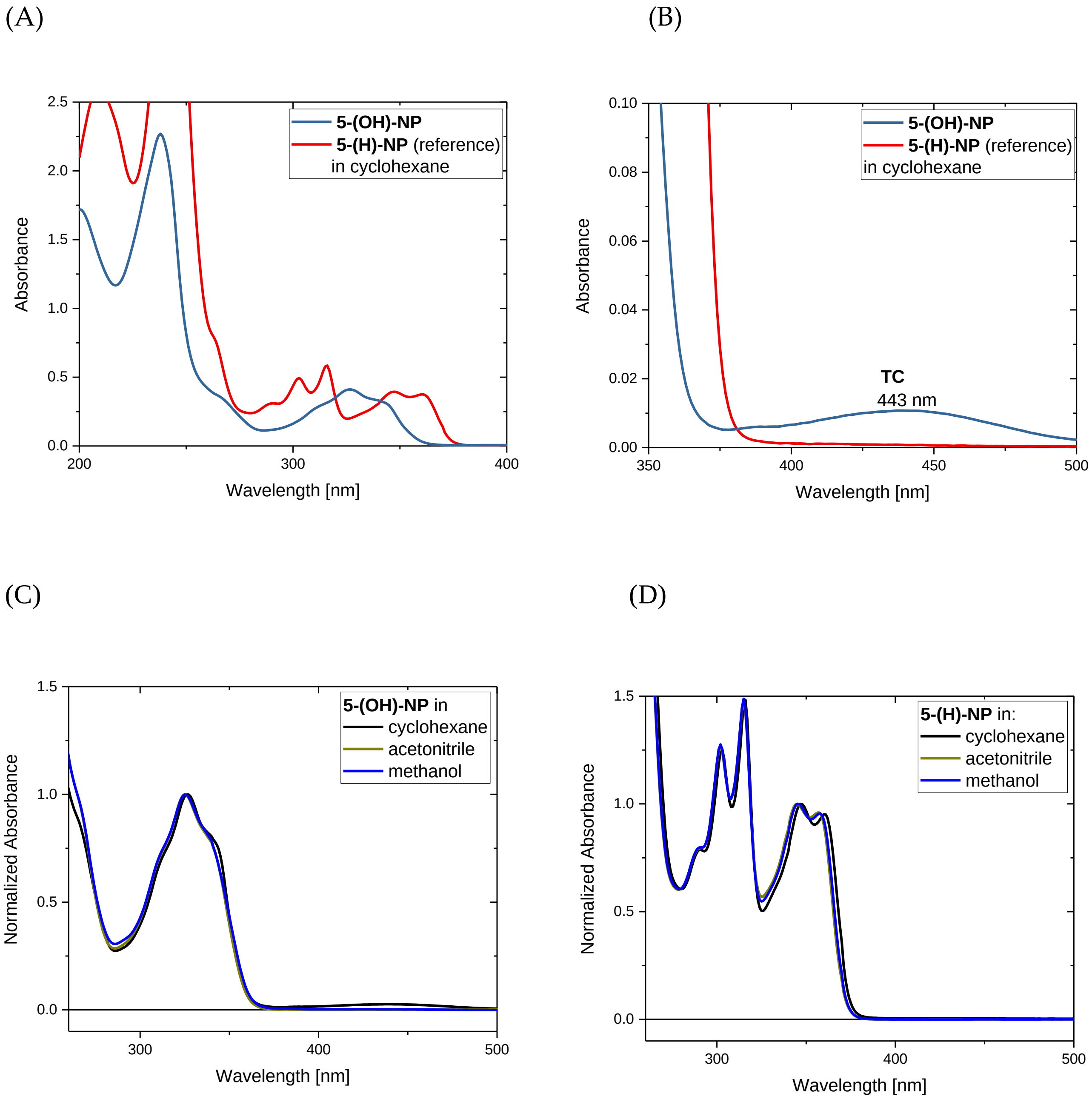

Figure S2. **TC** complexed with a single methanol (A) or acetonitrile (B) molecule optimized with the MP2/cc-pVDZ method. Among **MC** complexes **the MC1** is characterized by lowest energy due to the presence of two intramolecular hydrogen bonds. **AC1** is stabilized by ππ stacking interaction.

(A) **TC-methanol** complexes:

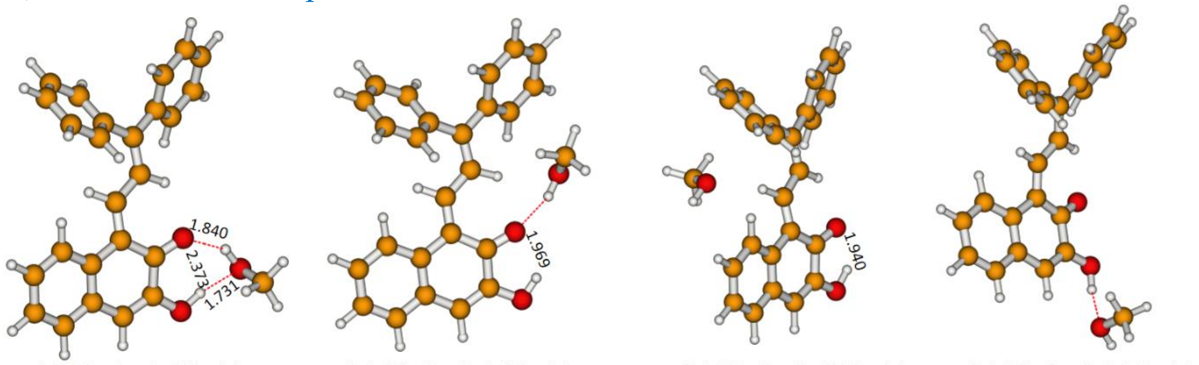

MC1, S_0 0.00 eV **MC2**, S_0 0.048 eV **MC3**, S_0 0.179 eV **MC4**, S_0 0.332 eV

(B) **TC-acetonitrile** complexes**:**

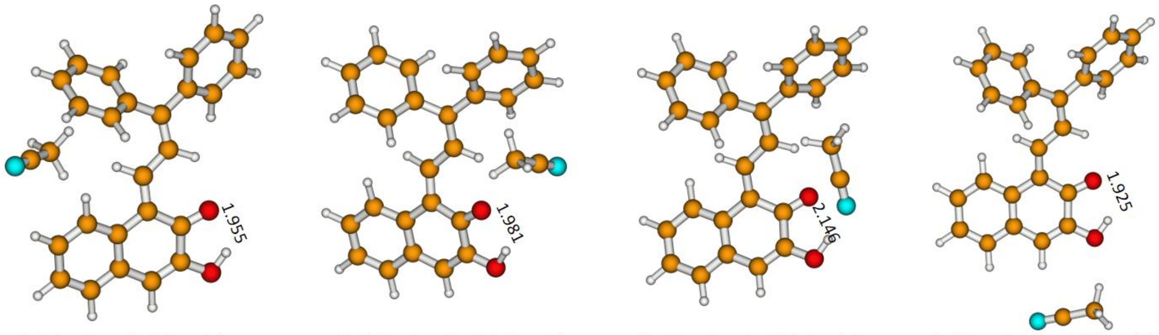

AC1, S_0 0.00 eV **AC2**, S_0 0.029 eV **AC3**, S_0 0.074 eV **AC4**, S_0 0.125 eV

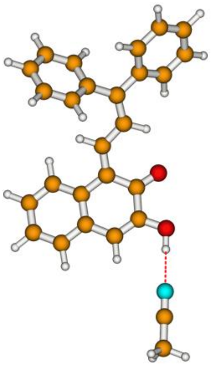

AC5, S_0 0.332 eV

Figure S3. Global analysis of data presented in Figures 3C and 3D. Note the drastically diminished amplitude of the **TT** absorption band from **5-(OH)-NP** in comparison to **5-(H)-NP**.

(A)

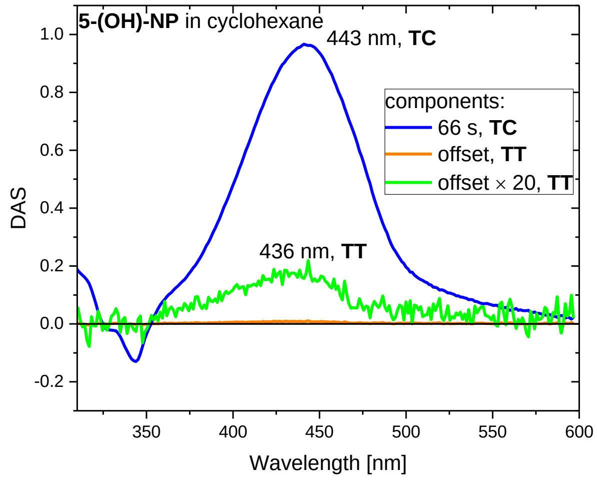

(B)

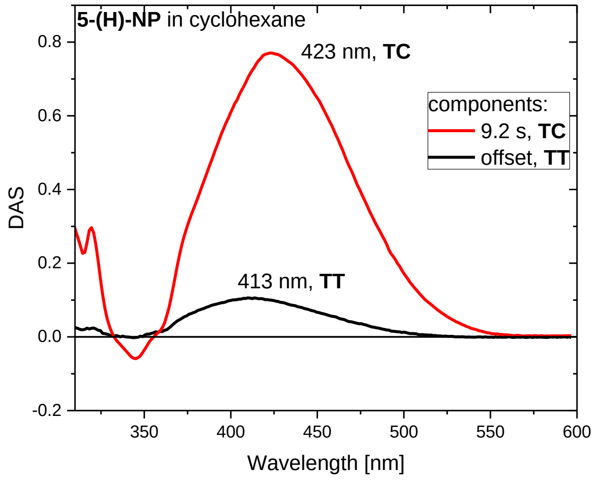

Figure S4. (A) and (B) Evolution of ΔA spectra after ceasing UV irradiation (4 mW/cm^2) at t = 0 s for **5-(OH)-NP** and **5-(H)-NP** in methanol at 21°C, respectively. Solutions were initially prepared with the same absorption at the excitation wavelength (λ = 340 nm). (C) and (D) Decay associated spectra retrieved from global analysis using mono-exponential model function.

(A)

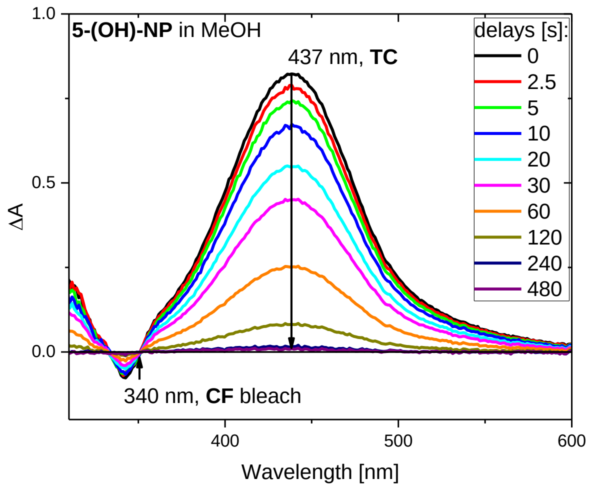

(B)

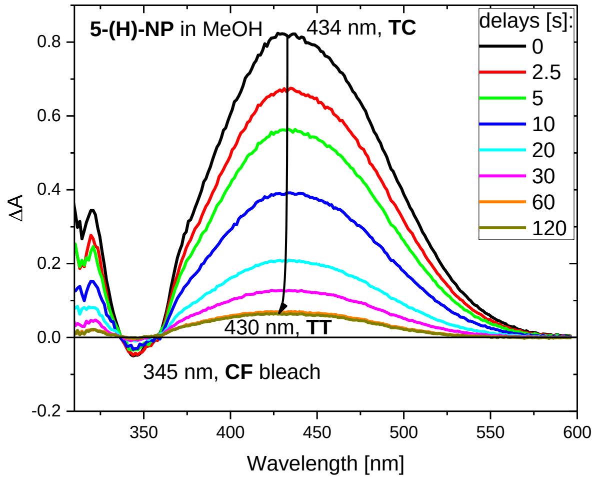

(C)

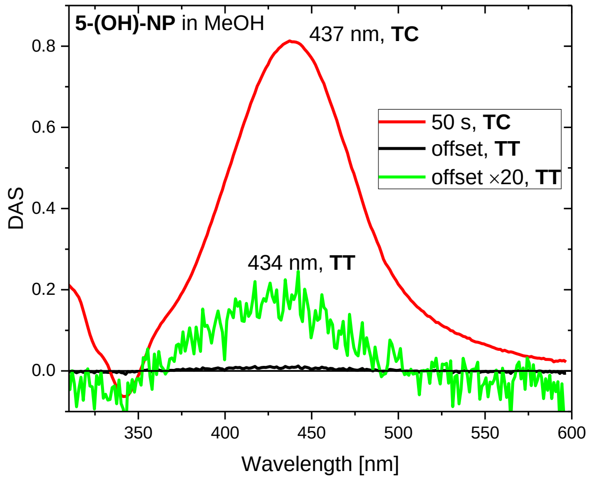

(D)

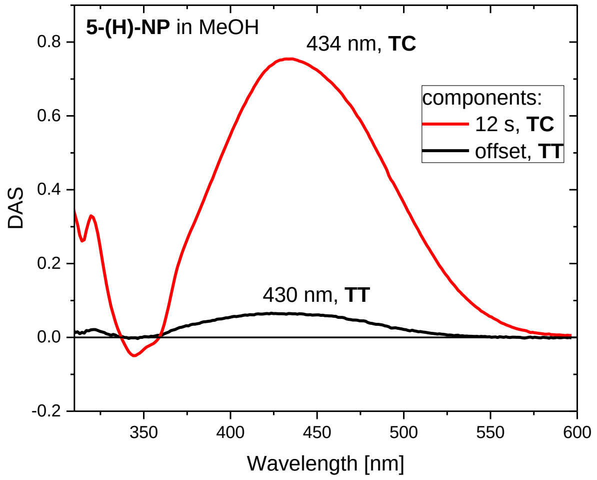

Figure S5. (A) Evolution of ΔA spectra after ceasing UV irradiation (3.2 mW/cm[2]) at t = 0 s for **5-(OH)-NP** in acetonitrile at 21°C. (B) Decay associated spectra retrieved from global analysis using a single-exponential model function.

(A) (B)

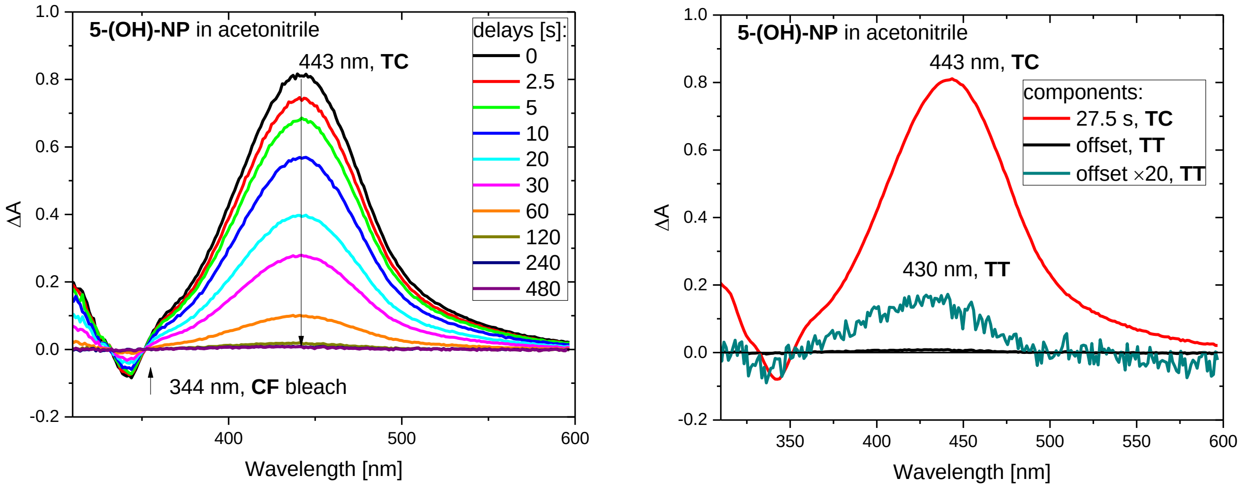

Figure S6. (A) UV-vis transient absorption spectra recorded for **TC** solution in methanol upon the laser pulse excitation at λ = 444 nm. PSS with a constant **TC** population was achieved by a continuous irradiation of **5-(OH)-NP** methanol solution with LED at λ = 340 nm. (B) Decay associated spectra obtained with global analysis using bi-exponential function.

(A) (B)

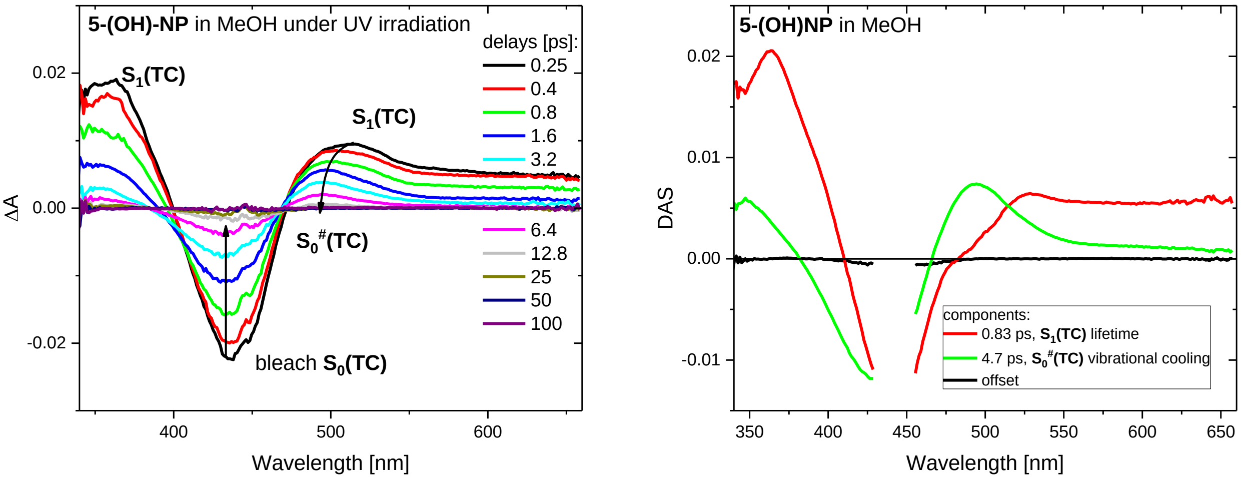

Figure S7. (A) UV-vis transient absorption spectra recorded for **TC** solution in acetonitrile upon the laser pulse excitation at λ = 444 nm. PSS with a constant **TC** population was achieved by a continuous irradiation of **5-(OH)-NP** acetonitrile solution with UV LED. (B) Decay associated spectra obtained with global analysis using bi-exponential function.

(A) (B)

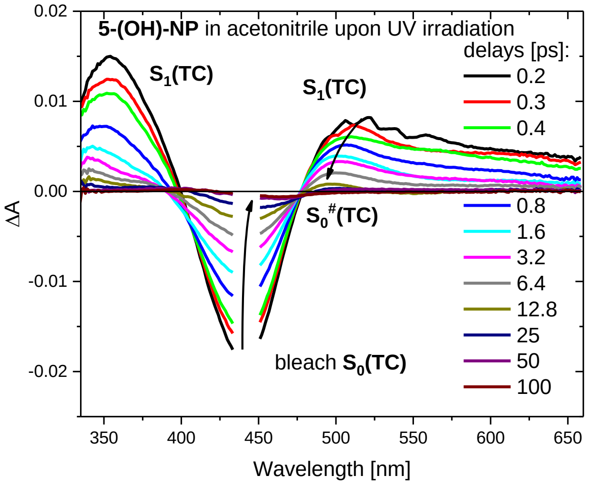

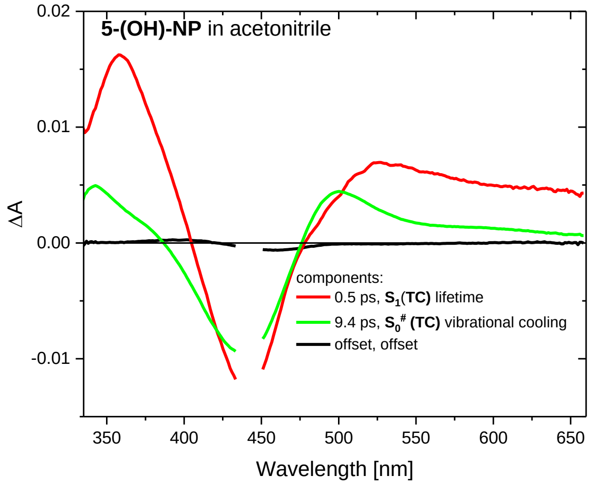

Table S1. Comparison of geometric parameters (bond lengths, in Å, dihedral angles), adiabatic energies (E_a, in eV) for the excited state (S_1) geometries optimized at the ADC(2)/cc-pVDZ level of theory. σ and τ denote $C_{14}{=}C_1{-}C_2{=}C_3$ and $C_5{-}C_{13}{-}C_{14}{=}C_1$ dihedral angles, respectively.

S_0- or S_1-State Form	R_1 $C_3{-}O_4$ [Å]	$θ_1$ $C_{13}{-}C_{14}{=}C_1{-}C_2$ [°]	$θ_2$ $C_1{-}C_2{=}C_3{-}C_{1'}$ [°]	σ [°]	τ [°]	R_2 $C_{13}{-}O_4$ [Å]	R_D $C_1{-}C_{14}$ [Å]	R_S $C_1{-}C_2$ [Å]	R_E $C_2{-}C_3$ [Å]	E_a [eV]
				**5-(OH)-NP (H-bonded)**						
S_0 ^{CF}	1.464	19.4°	79.5°	3.4°	173.6°	1.376	1.461	1.361	1.516	0.00
S_0 ^{INT}	2.789	18.5°	8.5°	41.8°	155.7°	1.248	1.390	1.443	1.382	0.638
S_0 ^{TC}	4.173	-1.5°	9.8°	-174.2°	-164.8°	1.248	1.387	1.436	1.385	0.407
S_1 ^{TC}	4.195	-39.2°	14.2°	179.1°	-176.7°	1.352	1.485	1.373	1.434	1.305

Table S2 Vertical excitation energy (*ΔE*^{VE}, in eV and λ_{abs} , in nm), oscillator strength (*f*), of the lowest singlet states for the equilibrium forms of the **5-(OH)-NP** and **5-(H)-NP** molecules calculated with the **ADC(2)/cc-pVDZ** method at the singlet excited state **TC** geometry optimized at the ADC(2)/**cc-pVDZ** theory level. Typically, theoretical calculated spectral positions of the maxima are overestimated by about 0.2 eV in comparison to experiment. Thus, we deduce that the positive initial band with maxima at 520 and 360 nm (**5-(OH)-NP** in cyclohexane, Figure 5) is not the **TC*-proton transferred** population. Note that such calculations reproduce satisfactorily the **TC*** absorption band for **5-(H)-NP** reported in Brazevic *et al*., ChemPhysChem 21, 1402–1407, (2020).

compound		ΔE^{VE}	λ_{abs}	*f*
**TC*-proton transferred 5-(OH)-NP**				
	$S_1 \rightarrow S_2(n\pi^*)$	1.24	1000	0.00008
	$S_1 \rightarrow S_3(\pi\pi^*)$	1.96	633	0.0104
	$S_1 \rightarrow S_4(\pi\pi^*)$	**3.20**	***388***	0.2906
	$S_1 \rightarrow S_5(\pi\pi^*)$	**3.28**	***379***	0.4077
	$S_1 \rightarrow S_6(\pi\pi^*)$	**3.70**	**335**	0.1109
	$S_1 \rightarrow S_7(\pi\pi^*)$	**3.86**	**322**	0.2021
**TC* 5-(H)-NP**				
	$S_1 \rightarrow S_2(n\pi^*)$	0.67	1855	0.0012
	$S_1 \rightarrow S_3(\pi\pi^*)$	2.30	540	0.0073
	$S_1 \rightarrow S_4(\pi\pi^*)$	**2.60**	**477**	0.4401
	$S_1 \rightarrow S_5(\pi\pi^*)$	3.23	385	0.0018
	$S_1 \rightarrow S_6(\pi\pi^*)$	3.31	375	0.0083
	$S_1 \rightarrow S_7(\pi\pi^*)$	**3.37**	**368**	0.0418
	$S_1 \rightarrow S_8(\pi\pi^*)$	**3.52**	352	0.0100
	$S_1 \rightarrow S_9(\pi\pi^*)$	**3.67**	**338**	0.0671
	$S_1 \rightarrow S_{10}(\pi\pi^*)$	3.69	336	0.0206

Table S3. Lifetime τ_{S1} of **TC** in the singlet excited state for **5-(OH)-NP** and **5-(H)-NP** (at 22 °C).

Compound	Solvent	τ_{S1}(TC), ps
5-(OH)-NP	cyclohexane	0.2
	acetonitrile	0.5
	methanol	0.8
5-(H)-NP	cyclohexane	0.8 ref [54]
	acetonitrile	0.35 ref [54]
	methanol	0.35
